# Role of Menstrual Bleeding Assessments in Sickle Cell Clinics

**DOI:** 10.1001/jamanetworkopen.2025.46345

**Published:** 2025-12-09

**Authors:** Gillian Rush, Rania E. Mohamed, Kimberly Moffatt-Bazile, Sri Lakshmi Jamalapur, Gianna G. Valenti, Bindu K. Sathi, Seethal Jacob, Layla N. Van Doren, Maria C. Velez, Ugochi Ogu, Gayle M. Smink, Corinna L. Schultz, Nicole DiVirgilio, Esteban Gomez, Maa-Ohui Quarmyne, Jennifer Light, Kalpna Gupta, Li Zhang, Elliott P. Vichinsky, Ward Hagar, Angela Rivers, Marsha Treadwell, John J. Strouse, Neha Bhasin

**Affiliations:** 1 Division of Hematology, Department of Pediatrics, University of California, San Francisco; 2 Division of Hematology, Department of Medicine, Duke University, Durham, North Carolina; 3 Division of Hematology and Oncology, Department of Pediatrics, Children’s Hospital of Michigan, Detroit; 4 Division of Hematology and Oncology, Department of Pediatrics, Valley Children’s Hospital, Madera, California; 5 Division of Hematology and Oncology, Department of Pediatrics, Riley Hospital for Children, Indianapolis, Indiana; 6 Division of Hematology, Department of Medicine, Yale University, New Haven, Connecticut; 7 Division of Hematology and Oncology, Department of Pediatrics, Manning Family Children’s Hospital, Louisiana State University Health Sciences Center, Shreveport; 8 Division of Hematology and Oncology, Department of Medicine, University of Tennessee Health Science Center, Knoxville; 9 Division of Hematology and Oncology, Department of Pediatrics, Penn State University, Hershey, Pennsylvania; 10 Division of Hematology and Oncology, Department of Pediatrics, Nemours Children’s Hospital, Wilmington, Delaware; 11 Division of Hematology and Oncology, Department of Pediatrics, Center for Inherited Blood Disorders, Orange, California; 12 Division of Hematology and Oncology, Department of Pediatrics, Phoenix Children’s Hospital, Phoenix, Arizona; 13 Division of Hematology and Oncology, Department of Pediatrics, University of Illinois Peoria; 14 Division of Hematology and Oncology, Department of Medicine, University of California, Irvine

## Abstract

**Importance:**

The interplay between sickle cell disease (SCD) and reproductive health remains understudied. Menstruation is particularly complex in individuals with SCD, who may have delayed puberty, early menopause, abnormal uterine bleeding, and overlapping SCD-related and menstruation-related pain.

**Objective:**

To assess menstrual patterns in female patients with SCD, these patients’ access to and use of contraceptive therapy, and clinician practices associated with reproductive health.

**Design, Setting, and Participants:**

This cross-sectional survey study was conducted in 13 outpatient SCD centers across the US. Female patients with SCD who had reached menarche 12 or more months prior to enrollment between March 1, 2022, and May 31, 2024, and were not pregnant or post menopausal were included. Clinicians involved in the care of enrolled participants were also surveyed on a voluntary basis.

**Main Outcomes and Measures:**

Age-appropriate menstrual bleeding questionnaires were used to assess menstrual patterns, and global health was evaluated using the Patient-Reported Outcomes Measurement Information System mental and physical health scales. Newly designed patient-facing surveys were used to assessed SCD health and contraceptive use. Clinician-facing surveys assessed participants’ clinical history and care plans after reviewing the patient surveys.

**Results:**

A total of 211 participants (mean [SD] age 23.7 [10.1] years) were enrolled. All patient-facing surveys with a corresponding clinician survey were completed for 183 participants (86.7%). Abnormal uterine bleeding was noted in 52 participants (24.6%) and was associated with a higher rate of hospitalizations (59.1% hospitalized in this group vs 34.5% in the non–abnormal uterine bleeding group). Most participants (134 of 208 [64.4%]) endorsed sickle cell pain with menses. Among these participants, 42 (36.5%) had no emergency department (ED) visits, 51 (44.3%) had 1 to 3 ED visits, and 22 (19.1%) had 4 or more ED visits in the 6 months prior to enrollment compared with those who did not endorse sickle cell pain associated with menstruation (38 [58.5%], 24 [36.9%], and 3 [4.6%], respectively). In addition, among the participants who endorsed sickle cell pain, 57 (49.6%) had 0 hospitalizations, 48 (41.7%) had 1 to 3 hospitalizations, and 10 (8.7%) had 4 or more hospitalizations compared with those who did not endorse sickle cell pain associated with menstruation (50 [76.9%], 14 [21.5%], and 1 [1.5%], respectively). There was minimal use of hormonal contraception (40 of 208 participants [19.2%]), and 46 participants (22.1%) reported that they had never heard of these medications. After reviewing the patient survey, clinicians reported several intended steps to improve reproductive health for their patients, including reproductive health education (131 participants [72.4%]), referral to a reproductive health specialist (90 participants [49.7%]), or workup for iron deficiency (29 participants [16.0%]) or an underlying bleeding disorder (21 participants [11.6%]). Three participating sites established new multidisciplinary clinics for reproductive health and hematology during the enrollment period.

**Conclusions and Relevance:**

In this multicenter survey study of female individuals with SCD, sickle cell pain with menses was prevalent, and hormonal contraceptive use was low. The findings suggest that brief assessments may facilitate dialogue between female patients with SCD and their sickle cell clinicians and lay the groundwork for improved reproductive health and medical management.

## Key Points

**Question:**

What is the association of reproductive health assessment among female patients with sickle cell disease (SCD) and menstrual-related sickle cell pain and hormonal therapies?

**Findings:**

In this survey study of 211 female patients conducted across 13 US SCD centers, high rates of menstrual-related SCD pain (64.0%) and low rates of contraceptive use (19.2%) were found. In 98.3% of instances, clinicians reported intended clinical actions based on patient survey responses.

**Meaning:**

These findings suggest that menstrual assessments in sickle cell clinics may improve attention and personalized care for female patients with SCD.

## Teaser

This survey study evaluates menstrual patterns among female patients with sickle cell disease, access to and use of contraceptive therapy, and their clinicians’ practices associated with reproductive health in the US.

## Introduction

Sickle cell disease (SCD) is an autosomal recessive disorder affecting 120 000 people in the US and 8 million people worldwide.^1,2^ Sickle cell disease causes chronic hemolysis, vaso-occlusive events (VOEs), pain, and end-organ damage, with high morbidity and mortality.^3^ Vaso-occlusive events can be triggered by cold exposure, stress, dehydration, and low oxygen saturation, but menstruation is often overlooked as a cause of VOE. With improvement in care, many individuals with SCD in the US are living through their reproductive years.^4^ However, reproductive health and its impact on overall health in SCD remains understudied.^3,5^

The quality of one’s menstruation has been associated with social well-being and can serve as a marker of reproductive function.^6^ Menstrual patterns may predict long-term health outcomes.^3^ Individuals with SCD experience delayed puberty and early menopause.^3,7,8^ In addition, menstruation is associated with pain, which may affect the VOE-related pain experience in female individuals with SCD. Recent studies have shown an association between menstruation and VOEs and a high incidence of abnormal uterine bleeding (AUB) in women with SCD.^3,9,10,11,12^ A longitudinal study showed that female individuals with SCD aged 10 to 39 years had higher rates of acute pain episodes than their male counterparts.^13^ Hormonal contraception might be an option to mitigate the effects of menstruation-associated VOEs and decrease AUB; however, there are limited data to guide the efficacy and safety of these therapies in SCD.

Our aim in this study was to assess menstrual patterns and contraceptive awareness, access, and use and to identify clinician practices associated with reproductive health for female individuals with SCD. These findings may offer a critical foundation for building multidisciplinary, patient-centered reproductive care that addresses the distinct needs of persons with SCD.

## Methods

### Participating Sites

This cross-sectional survey study of female individuals with SCD and their health care practitioners was conducted across 13 sickle cell centers. The study was approved by the University of California, San Francisco (UCSF) Institutional Review Board. All 12 additional participating sickle cell sites (4 lifespan, 2 adult, and 6 pediatric) obtained local institutional review board approval, with UCSF (a lifespan center) serving as the coordinating site. All participants provided informed consent or assent. Data use agreements were executed. The study followed the American Association for Public Opinion Research (AAPOR) reporting guideline.

### Inclusion and Exclusion Criteria

The study was open to enrollment from March 1, 2022, to May 31, 2024. Menstruating female patients aged 12 to 54 years with SCD (all genotypes) who experienced menarche more than 12 months prior to enrollment and who had menstrual bleeding at least once in the past 12 months were recruited. We did not collect race and ethnicity data, as we did not believe that they were relevant to the aims of this study. Patients who were pregnant, postmenopausal, and unable to speak and/or read English were excluded.

### Study Documents

Patient-facing surveys required approximately 15 minutes to complete. Clinician surveys were optional and requested after the clinician reviewed the patient surveys.

#### Validated Surveys

Age-appropriate validated menstrual bleeding questionnaires (MBQs) were used to evaluate menstrual bleeding patterns. The adult MBQ (eAppendix 1 in Supplement 1) was used for participants aged 18 years or older, and the adolescent MBQ (eAppendix 2 in Supplement 1) was used for participants younger than 18 years.^14,15^ An MBQ score of at least 24 (on a scale from 0-75) was used to define AUB.^16^ Global health was evaluated using the 4-item Patient-Reported Outcomes Measurement Information System (PROMIS) Scale, version 1.2 Global Mental 2a and Global Physical 2a (eAppendix 3 in Supplement 1).

#### Newly Created Surveys

The study-specific patient survey (eAppendix 4 in Supplement 1) and clinician survey (eAppendix 5 in Supplement 1) were developed in conjunction with the Foundation for Women and Girls With Blood Disorders SCD Learning Action Network through monthly subcommittee discussions between March 1, 2021, and March 31, 2022, to assess participant health care use, clinical data, and access to and use of medications. Clinician actions based on patient survey responses were also collected.

### Data Management

Participants were approached and consented during routine sickle cell clinic visits. They completed the MBQ, PROMIS, and patient survey either remotely or on site. All data were entered into a central database hosted on UCSF’s Research Electronic Data Capture platform (REDCap; Vanderbilt University). Clinician surveys were encouraged but not required.

### Statistical Analysis

Categorical variables were summarized using counts with percentages, and between-group comparisons were performed using the χ^2^ test. Continuous variables were described by means with SDs and ranges, with between-group comparisons by *t* test (if more than 2 groups, analysis of variance was used). If the continuous variables did not hold to the normality assumption, then the median and IQR were used to describe the data, and nonparametric tests (Wilcoxon rank sum test or Kruskal-Wallis analysis of variance) were used to compare the variables across groups. Statistical significance was considered at *P* < .05. No multiple testing adjustment was performed. All statistical analyses were performed using R, version 4.3.2 (R Foundation for Statistical Computing).

## Results

### Patient Demographics and Survey Completion

A total of 211 patients were enrolled and completed the MBQs (mean [SD] age, 23.7 [10.1] years; 148 with sickle cell hemoglobin [HbS] S [70.1%], 38 with HbSC [17.9%], 6 with HbSβ0 [2.8%], 18 with HbSβ+ [8.5%], and 1 with other [0.5%] genotype). All surveys completed based on genotype are shown in Table 1. All patient-facing surveys with a corresponding clinician survey were completed for 183 participants (86.8%). Mean (SD) age of menarche was 12.1 (1.2) years (range, 9.0-15.0 years) among 71 adolescent patients, as it was only assessed in the adolescent MBQ and was not statistically different among the genotypes (Table 1).

**Table 1.** Participant Surveys Completed and MBQ Scores Based on Genotype

Variable	Patients by genotype, No. (%)						*P* value
	Overall	HbSS	HbSC	HbSβ0	HbSβ+	Other	
Patients, No. (%)	211 (100)	148 (70.1)	38 (17.9)	6 (2.8)	18 (8.5)	1 (0.5)	NA
Age, mean (SD), y	23.7 (10.1)	23.4 (9.87)	23.8 (10.0)	26.7 (10.4)	25.5 (11.9)	15 (NA)	.75
Age of menarche (n = 71), mean (SD), y	12.1 (1.2)	12.3 (1.2)	11.4 (1.0)	12.0 (NA)	11.8 (1.1)	14 (NA)	.09
MBQs completed	211 (100)	148 (100)	38 (100)	6 (100)	18 (100)	1 (100)	NA
MBQ score, mean (SD)	17.5 (10.4)	16.9 (9.6)	18.7 (12.5)	18.7 (13.4)	18.8 (11.4)	18 (NA)	.86
AUB^a^	52 (24.6)	31 (20.9)	12 (31.6)	2 (33.3)	7 (38.9)	0 (NA)	.33
PROMIS survey completed	181 (85.8)	126 (85.1)	33 (86.8)	5 (83.3)	16 (88.9)	1 (100)	>.99
Patient survey completed	208 (98.6)	146 (98.6)	38 (100)	6 (100)	17 (94.4)	1 (100)	.42
Clinician survey completed	183 (86.7)	131 (88.5)	32 (84.2)	6 (100)	13 (72.2)	1 (100)	.26

Abbreviations: AUB, abnormal uterine bleeding; HbS, sickle cell hemoglobin; MBQ, menstrual bleeding questionnaire; NA, not applicable; PROMIS, Patient-Reported Outcomes Information System.

^a^ The MBQ screening cutoff of 24 or higher (on a scale of 0-75) was used to diagnose AUB.

### Results for Validated Measures

**Figure 1.**
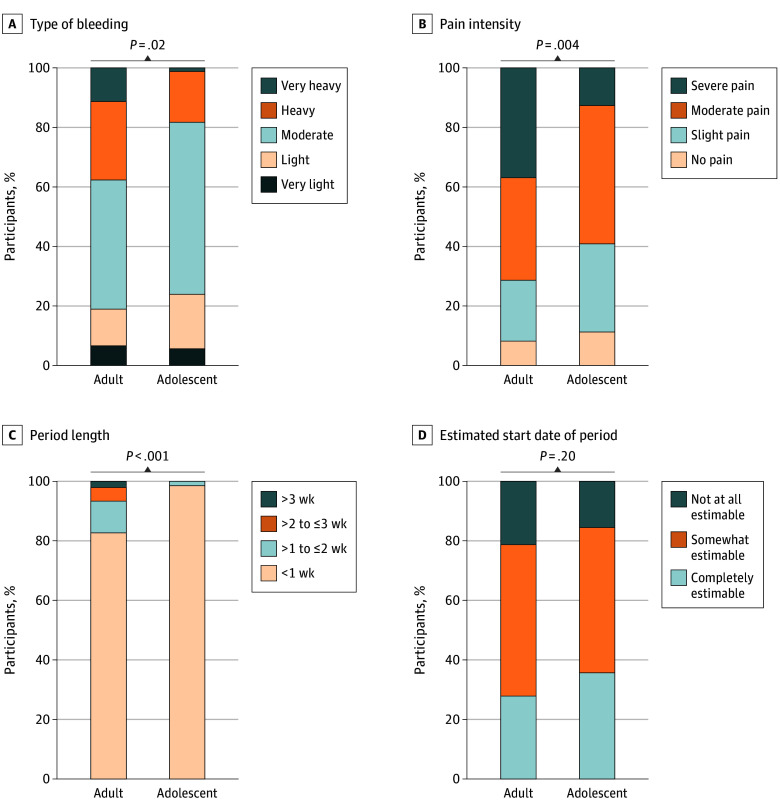
Key Measurements From the Menstrual Bleeding Questionnaire

The mean MBQ score was 17.5 (SD, 10.4) (Table 1) and not significantly different between adolescents and adults. Fifty-two participants (24.6%), including 41 of 141 adults (29.1%) and 12 of 71 adolescents (16.9%), had AUB based on their MBQ score of 24 or greater (*P* = .08). The MBQ scores and AUB were not significantly different among the genotypes (Table 1); however, AUB was associated with higher rates of hospitalizations in the past 6 months (18 of 44 participants [40.9%] with 0 hospitalizations, 24 of 44 [54.5%] with 1-3 hospitalizations, and 2 of 44 [4.5%] with ≥4 hospitalizations vs 91 of 139 [65.5%] with 0 hospitalizations, 38 of 139 [27.3%] with 1-3 hospitalizations, and 10 of 139 [7.2%] with ≥4 hospitalizations in the non-AUB group) (*P* = .004) (Table 2). Key measurements in the MBQ, including type of bleeding, pain intensity, period length, and estimated start date of period are summarized in Figure 1, showing significant differences between adolescents and adults. No significant differences among the genotypes were observed.

**Table 2.** Association of AUB With Health Care Use

Variable	Overall (N = 183)	MBQ <24 (n = 139)	AUB (n = 44)	*P* value
ED visits in the past 6 mo				
0	81 (44.3)	66 (47.5)	15 (34.1)	.29
1-3	75 (41.0)	54 (38.8)	21 (47.7)	
≥4	27 (14.8)	19 (13.7)	8 (18.2)	
Hospitalizations in the past 6 mo				
0	109 (59.6)	91 (65.5)	18 (40.9)	.004
1-3	62 (33.9)	38 (27.3)	24 (54.5)	
≥4	12 (6.6)	10 (7.2)	2 (4.5)	

Abbreviations: AUB, abnormal uterine bleeding; ED, emergency department; MBQ, menstrual bleeding questionnaire.

The PROMIS surveys were completed by 181 participants (85.8%) (Table 1). The scores were significantly lower than the reference population mean of 50,^17^ even though they were within 1 SD of the reference population. The mean (SD) *t* scores for adult participants were 45.9 (7.7) for physical health and 48.4 (8.8) for mental health. The mean (SD) *t* scores for adolescent participants were 47.8 (8.8) for physical health and 47.8 (8.8) for mental health. The PROMIS scores were not significantly different between adolescent and adult participants for physical health or mental health.

### Results for Newly Created Surveys

Study-specific patient surveys were completed by 208 participants (98.6%). When asked, “Do you experience sickle cell pain around your menstrual cycle,” 134 of 208 participants (64.4%) responded yes, with no significant difference in responses among genotypes. Those who responded yes had higher MBQ scores (mean [SD], 19.6 [11.2] vs 13.4 [6.9] responding no; *P* < .001). Among participants who responded yes, 124 completed the questions about pain severity in the MBQ, with 7 (5.6%) reporting no pain, 22 (17.7.8%) reporting slight pain, 46 (37.1%) reporting moderate pain, and 49 (39.5%) reporting severe pain with menstruation. Among participants who responded no (n = 72), 64 completed the questions about pain severity in the MBQ, with 10 (15.6%) reporting no pain, 24 (37.5%) reporting slight pain, 26 (40.6%) reporting moderate pain, and 4 (6.2%) reporting severe pain with menstruation (*P* < .001). In addition, those who responded yes had a greater number of emergency department (ED) visits (*P* = .003) and hospitalizations (*P* = .001) than those who responded no. More adults (96 of 137 [70.1%]) than adolescents (38 of 69 [55.1%]) responded yes (*P* < .03) to this question.

In terms of medications, most patients reported taking disease-modifying therapies for at least 6 months prior to survey completion, including 119 of 154 patients with HbSS and HbSB0 taking hydroxyurea (77.3%), 8 of 208 (3.8%) taking crizanlizumab, 24 of 208 (11.5%) taking voxelotor, and 11 of 208 (5.3%) taking l-glutamine.

**Figure 2.**
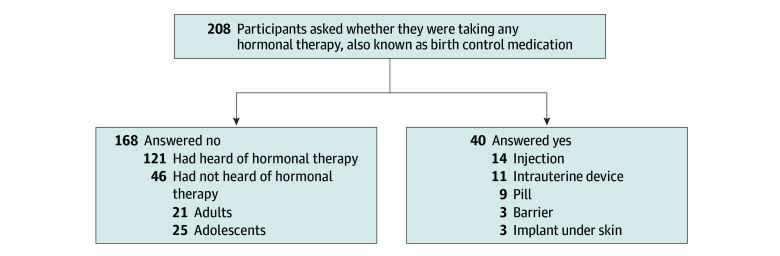
Patient-Reported Use of Hormonal Therapy

Hormonal contraception use was reported by 40 of 208 participants (19.2%) (Figure 2) with 15 of 40 (37.5%) reporting taking these medications for period-related pain or sickle cell pain and 11 of 40 (27.5%) for pregnancy prevention. Most participants receiving these therapies (19 of 40 [47.5%]) reported receiving them from a gynecologist (21 of 40 [52.5%]) or a combination of primary care practitioners, adolescent practitioners, hematologists, or elsewhere. Participants not taking hormonal contraception (168 [80.8%]) were asked whether they had heard of hormonal therapy, also known as birth control. Within this group, 46 (27.3%), including 21 adults (45.7%) and 25 adolescents (54.3%), reported that they had never heard of hormonal therapy (Figure 2).

Clinician surveys were completed by 21 clinicians. Medians for laboratory data are shown in eTable 1 in Supplement 1. A ferritin level of less than 50 ng/mL (50 µg/L) was noted in 13 participants (12.3%). Low hemoglobin and high total bilirubin, reticulocyte counts, and absolute neutrophil counts were not associated with higher MBQ scores or endorsement of SCD pain around menstruation. However, lower hemoglobin was associated with increased ED visits and admissions. The mean (SD) hemoglobin was 9.56 (1.50) g/dL in participants with 0 ED visits, 9.05 (1.45) g/dL in participants with 1 to 3 ED visits, and 8.75 (1.49) g/dL in participants with 4 or more ED visits (*P* = .03) (to convert to g/L, multiply by 10). The mean (SD) hemoglobin was 9.44 (1.40) g/dL in participants with 0 admissions, 9.01 (1.43) g/dL in participants with 1 to 3 admissions, and 8.37 (1.05) g/dL in participants with 4 or more admissions (*P* = .04).

Given the risk of thrombosis with SCD and estrogen-containing hormonal contraceptives, we inquired about the history of venous thromboembolism and arterial thromboses. Of 183 participants, 27 (14.8%) who had a clinician survey completed had a history of venous thromboembolism (24 of 27 [88.9%]), stroke (5 of 27 [18.5%]), or both (2 of 27 [7.4%]).

**Figure 3.**
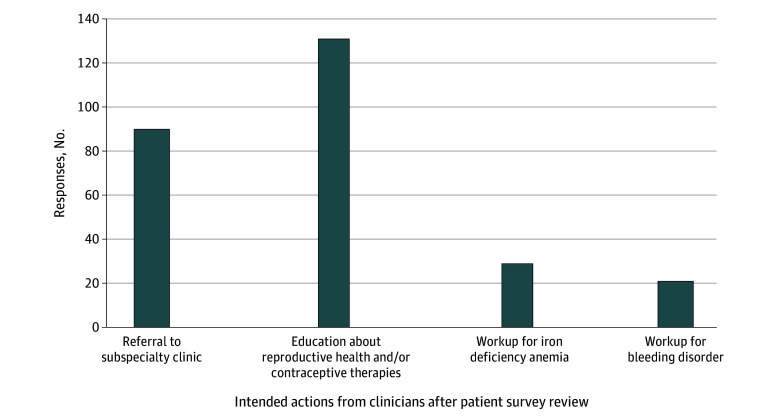
Intended Actions From Clinicians After Review of Patient Surveys

When asked what next steps clinicians were planning to take in response to patient survey results, most planned an intervention. The clinician selections are summarized in Figure 3, with at least 1 selection made for 178 of 183 participants (97.3%). Providing education was selected for 131 participants (72.4%), referring to a different health specialist for women’s health for 90 participants (49.7%), performing an iron deficiency workup for 29 participants (16.0%), and performing a bleeding disorder workup for 21 participants (11.6%). Participants with higher MBQ scores were significantly more likely to be selected for intended referrals to obstetrics and gynecology, with mean (SD) scores for those referred of 22.2 (10.8) vs 15.2 (9.3) for those not referred (*P* < .001). In addition, participants were more likely to be selected for intended referrals to other women’s health specialists if they had higher MBQ scores, with a mean (SD) score for those referred of 21.1 (9.5) vs 16.7 (10.4) for those not referred (*P* = .02). They were also more likely to undergo an intended iron deficiency workup (*P* = .004) or bleeding disorder workup (*P* < .001), with mean (SD) MBQ scores of 22.5 (11.5) and 25.2 (7.7), respectively, compared with 16.8 (9.4) and 16.5 (SD 9.9) among those who did not undergo these workups, respectively. Clinicians also intended to refer to obstetrics and gynecology when patients endorsed sickle cell pain around their menstrual cycle (55 of 134 participants [41.0%] referred for pain around their menstrual cycle vs 13 of 72 participants [18.1%] not reporting sickle cell pain around their menses) (*P* = .001).

At the completion of study enrollment, all site principal investigators were sent a closeout survey about reproductive health clinics for their patients preceding and after participation in this study (eAppendix 6 in Supplement 1). Four of the 13 participating sites (30.8%) had an established multidisciplinary clinic (hematologist and reproductive health specialist) for female patients with SCD at study startup. By the end of the study, an additional 3 multidisciplinary clinics were established (eTable 2 in Supplement 1).

## Discussion

This survey study found a high rate of menstruation-related pain in female patients with SCD, which was associated with high health care use and minimal use of hormonal contraception. We found that clinician awareness about the patients’ reproductive health through validated and study-specific questionnaires was associated with important intended actions by the clinicians to improve the health of their patients. The results of our study highlight that SCD and reproductive health may be intertwined and need to be regularly reviewed to improve the overall health of patients.

Patients with SCD have increased health care use and mortality.^18,19^ Participants in this study who endorsed menstruation-related sickle cell pain had a higher number of ED visits and hospitalizations. In addition, AUB was associated with a higher rate of hospitalizations. These findings are supported by a recent study that showed that female individuals of reproductive age had higher health care use than male individuals with SCD.^20^ These data suggest that sickle cell pain triggered by menses and AUB may have a direct impact on health care use, thereby affecting morbidity and mortality among female individuals with SCD.

Several studies have shown that women with SCD have pain associated with their menstrual cycles, but its mechanisms are poorly understood.^3,9,10,11,12^ Our study confirmed this association with 64% of participants reporting sickle cell pain associated with menstrual cycles, which is much higher than 39.3% reported in a recent study of 178 women primarily from Ghana.^11^ Why we had a higher number of participants reporting sickle cell pain related to menstruation compared with this earlier study is unclear. A possible explanation may be a difference in patient perception of pain in the different populations or the age and genotype differences of the participants enrolled in the 2 studies. High MBQ scores were significantly correlated with reported SCD pain related to menstrual cycles in our study. These results highlight that menstrual pain and sickle cell pain may be interconnected rather than isolated entities, even though patient perceptions of menstrual pain and sickle cell pain are different.^12^ We noted that key MBQ questions had different responses between adolescent and adult participants (Figure 1), which are concerning for the evolution of menstrual issues in women with SCD and need to be studied further. Whether end-organ dysfunction, including uterine and ovarian ischemia related to anemia and vasculopathy, prostaglandin release, and hormonal changes, in aging women with SCD could be playing a role needs further investigation to fully understand the mechanisms of pain and menstrual issues in adolescents and adults with SCD.

The mean age of menarche for participants with SCD in this study was 12.1 years but was only available for 71 (33.5%). This age was very similar to the average age of menarche for all female individuals in the US of 12.8 years.^21,22^ This finding contrasts with prior studies reporting delayed ages of menarche for female individuals with SCD.^3,9^ We estimate that the normal age of menarche in our study may be related to our small sample size, different genotype distribution among our participants compared with prior studies, exposure to disease-modifying therapies, or differences in age of menarche among different countries. In this study, AUB was noted in 24.6% of participants, which is higher than AUB noted in the general US population of 16.4%, even though the methods used to define AUB in these studies were different.^23^ The prevalence of AUB may be higher because of the underlying pain experience of patients with SCD compared with the general population. Given that AUB in our study was defined by results from the MBQ, a tool not designed for women with SCD, using the MBQ to define AUB in women with SCD has its limitations. Better methods to assess AUB in women with SCD may be needed in the future.

The effects of hormonal contraception on reproductive health and pain in female individuals with SCD lacks evidence. Despite the potential benefits of contraceptives, their use in SCD is nuanced. As a result, hormonal contraception is minimally prescribed and used by female individuals with SCD.^24,25,26,27^ Sickle cell disease and estrogen-containing contraceptives are associated with an increased risk for venous thromboembolism.^28,29^ However, all studies evaluating the use of progestin-only contraceptives in persons with SCD have found no increased risk of thrombosis.^30^ Depo medroxyprogesterone acetate is the only hormonal contraception medication found to decrease VOE in SCD.^31^ Only 19% of our study participants were taking any hormonal contraception, and 22% of the participants, including 21 adults and 25 adolescents, reported that they had never heard of these medications. Whether minimal use and knowledge about these medications among female individuals with SCD is related to patient-, community-, or clinician-related factors is unclear.^30^ Most participants (91.4%) were not prescribed these medications through their sickle cell practitioners, with 51.4% receiving them from gynecologists and 40.0% from other sources, who may or may not be as informed about the risks and benefits of these therapies for patients with SCD. Given the complexity of hormonal contraception use in SCD, a collaboration between hematologists and reproductive specialists is necessary to maximize benefits of hormonal contraception while minimizing risks associated with these therapies. In addition, further studies that assess the effects of these therapies on pain, health care use, and thrombosis in female individuals with SCD are needed so that both clinicians and patients can make informed decisions.

The association of SCD with our patients’ physical and mental health is consistent with findings from other studies, as evidenced by significantly lower PROMIS scores. In a prior study, PROMIS scores worsened as pain levels increased, showing the influence of any pain, including menstrual pain, may have on the health-related quality of life of individuals with SCD.^32^

Our study aimed to characterize the intended actions of clinicians based on patient survey responses. Given that 86% of participants had an optional clinician survey completed, a high interest in improving reproductive health among our study sites was evident. Clinicians intended to discuss reproductive care with patients in more than 70% of cases, indicating their willingness to engage on this topic themselves when given the right tools. Additionally, 140 entries were selected by clinicians as intended actions once they identified an issue, including workup for bleeding disorders, iron deficiency, or a referral to a reproductive health specialist (Figure 3). These steps may not have been selected as intended actions without the discussion generated by the surveys. These results highlight how simple measures to assess reproductive health in sickle cell clinics may influence clinicians’ intent to address or treat these issues. We therefore propose an annual menstrual history screening for all women with SCD with the tools used in this study and the creation of multidisciplinary clinics with hematologists and reproductive health specialists collaborating to improve the overall health of individuals with SCD. Site principal investigators’ responses to the closeout survey regarding reproductive health clinics at their sites preceding and following participation in this study suggest that clinicians acknowledge the importance of interdisciplinary reproductive and SCD care.

### Limitations

This study had several limitations. Although it captured data from 13 sickle cell centers in the US that were already involved in the Foundation for Women and Girls With Blood Disorders SCD Learning Action Network, the findings lack generalizability to all sickle cell clinics and patients with SCD in the US. The MBQ was used as a tool to assess for AUB, but it is not a tool designed to assess AUB for female patients with SCD specifically. Age of menarche was limited in our cohort and may need to be reviewed in a larger cohort of patients with SCD in the US. As a survey-based study, we cannot determine whether intended clinician actions were later completed. Data on hormonal contraception and disease-modifying therapy use were self-reported, so we cannot be certain about adherence and response to therapies and what participants perceived as hormonal contraceptive therapies based on our results.

## Conclusions

This survey study of female individuals with SCD found that sickle cell pain and AUB are prevalent issues associated with quality of life and increased health care use. We also confirmed that the use of and education about hormonal contraceptive therapies among female patients with SCD is minimal. Through this study, we hope that by facilitating a dialogue about reproductive health between sickle cell clinicians and their patients that further steps and research will be taken to improve reproductive health in individuals with SCD.

## Notes

Supplement 1. **eAppendix 1.** Adult MBQ **eAppendix 2.** Adolescent MBQ **eAppendix 3.** Short PROMIS, Version 1.2 Physical Health and Short PROMIS, Version 1.2 Mental Health Scales **eAppendix 4.** Patient Survey **eAppendix 5.** Clinician Survey **eAppendix 6.** Closeout Survey for Participating Sites **eTable 1.** Clinical Laboratory Values Based on Genotype **eTable 2.** Site-Specific Multidisciplinary Clinic Services

Supplement 2.
Data Sharing Statement
