# Supplementary material for: Role of Menstrual Bleeding Assessments in Sickle Cell Clinics
Source: JAMA Netw Open. 2025 Dec 9;8(12):e2546345. doi: 10.1001/jamanetworkopen.2025.46345 (PMC12690426; doi:10.1001/jamanetworkopen.2025.46345)
Supplement: Supplement 1. — eAppendix 1. Adult MBQ eAppendix 2. Adolescent MBQ eAppendix 3. Short PROMIS, Version 1.2 Physical Health and Short PROMIS, Version 1.2 Mental Health Scales eAppendix 4. Patient Survey eAppendix 5. Clinician Survey eAppendix 6. Closeout Survey for Participating Sites eTable 1. Clinical Laboratory Values Based on Genotype eTable 2. Site-Specific Multidisciplinary Clinic Services [file jamanetwopen-e2546345-s001.pdf]

Supplemental Online Content

Rush G, Mohamed RE, Moffatt-Bazile K, et al. Role of menstrual bleed assessments in sickle cell clinics. *JAMA Netw Open*. 2025;8(11):e2546345.
doi:10.1001/jamanetworkopen.2025.46345

eAppendix 1. Adult MBQ

eAppendix 2. Adolescent MBQ

eAppendix 3. Short PROMIS, Version 1.2 Physical Health and Short PROMIS, Version 1.2 Mental Health Scales

eAppendix 4. Patient Survey

eAppendix 5. Provider Survey

eAppendix 6. Closeout Survey for Participating Sites

eTable 1. Clinical Laboratory Values Based on Genotype

eTable 2. Site-Specific Multidisciplinary Clinic Services

This supplemental material has been provided by the authors to give readers additional information about their work.

eAppendix 1. Adult MBQ

Adult Menstrual Bleeding Questionnaire
Patient ID # __________
Date of survey ______________(Date/Month/Year)

These questions ask for details about your period. Periods can be different from month to month. Please make sure you read all of the options. For this questionnaire, period refers to any bleeding that you have from your vagina, even if it is irregular.

Some of the questions may sound similar. Just read through each question carefully and give your best answer.

You may have other medical problems that could affect your answers. Please try to focus on questions and answers **ONLY** as they relate to your period.

During the past month, did you have ANY bleeding?

Yes.. [ ]

No... [ ]

If you answered "YES" continue to question 1.
If you answered "NO" skip to question 12.

1. During the past month, how would you describe your periods?

Very Light.. [ ]

Light.. [ ]

Moderate.. [ ]

Heavy... [ ]

Very Heavy.. [ ]

Instructions for questions 2, 3, and 4.

"High absorbency" sanitary products mean any type of tampon or a pad that is NOT a thin pantyliner.

"Soaked" means completely or almost completely stained and filled with blood.

2. On your heaviest day of bleeding during the past month, how many high absorbency sanitary products did you soak (either completely or almost completely)?

© 2025 Rush G et al. *JAMA Network Open*.

0…………………………………………………..…………………………….………… [ ]
1-4…………………………………….…………………………………………….......... [ ]
5-8……………………………………….........................……………….………… [ ]
9-12…………………………………………………….…………………………………… [ ]
13-16………………………………….…………………………………………........... [ ]
More than 16……………………………………................................……………. [ ]

3. During the past month, how often did you need to wear either an incontinence brief or more than one high absorbency sanitary product (either more than one pad, a pad and a tampon, more than one tampon) at a time to contain your bleeding?

Never…………………………………………………..…………………………………… [ ]
1-3 times……………………………….…………………………………………………… [ ]
4-6 times……………………………………………...........................………………… [ ]
7-10 times………….……………………………………………………….……………... [ ]
11 times or greater…………..…………………………………………….……………. [ ]

4. During the past month, how many times have you had an episode of bleeding that soaked through your "outer" clothes (pants, skirt, dress)?

Never…………………………………………………..…………………………………… [ ]
1-3 times……………………………….…………………………………………………… [ ]
4-6 times……………………………………………...........................………………… [ ]
Greater than 6 times………………………………….…………………….…………... [ ]

5. During the past month, how many times did you need to get out of bed in the middle of night (or during sleep hours) to change your sanitary products?

Never…………………………………………………..…………………………………… [ ]
1-3 times……………………………….…………………………………………………… [ ]
4-6 times……………………………………………...........................………………… [ ]
7-10 times………….……………………………………………………….……………… [ ]
11 times or greater…………..…………………………………………….……………. [ ]

6. During the past month, how many times did you pass blood clots (clumps of blood)?

Never…………………………………………………..…………………………………… [ ]
1-3 times……………………………….…………………………………………………… [ ]
4-6 times……………………………………………...........................………………… [ ]

© 2025 Rush G et al. *JAMA Network Open*.

Greater than 6 times…………………………………………………………….……….…... [ ]

7. During the past month, how often did passing blood clots (clumps of blood) stain your clothing?

Never…………………………………………..……………………………………. [ ]
1-3 times…………………………………………………………………………… [ ]
4-6 times……………………………………………..............................………………… [ ]
Greater than 6 times………………………………………………………………. [ ]

8. Please fill in the following statement about pain related to your period. During the past month, my period was associated with…

No pain……..……………………………………………..………………………………. [ ]
Slight pain………………………………………………..………………………………. [ ]
Moderate pain…………………………….…………………………………….……….. [ ]
Severe pain……………………………………………...…………………….…………. [ ]

9. During the past month, how many weeks did your periods last?

1 week or less out of 4 weeks…………………………………………..…………… [ ]
More than 1 week, less than 2 weeks out of 4 weeks…..………….……………………….. [ ]
More than 2 weeks, less than 3 weeks out of 4 weeks…..……………..……………………. [ ]
More than 3 weeks out of 4 weeks…..……………………………..…………………….. [ ]

10. During the past month, on how many days do you think your work at your job or school suffered because you were bleeding?

I am currently not working outside home or going to school………………………...…… [ ]
Never, my bleeding does not affect my work..…………………………………………….. [ ]
1-3 days……………………….…………………………………………………..…….…..... [ ]
4-8 days…………………………………………...........................…….……………..... [ ]
9-12 days…………………………………………………………………………………... [ ]
13 days or more…………………………..........................……………………… [ ]

11. During the past month, on how many days did you miss work or school because you were bleeding?

I am currently not working outside of the home……………………..………………………. [ ]
Never, my bleeding does not affect my work schedule…………………………………... [ ]

© 2025 Rush G et al. *JAMA Network Open*.

1-3 days………………………………………………………………………………..……….. [ ]

4-8 days……………………………………………...........................………………… [ ]

9-12 days…………………………………………………………………………………... [ ]

13 days or more…………………………….......................………………… [ ]

12. During the past month, on how many days did you avoid family activities (grocery shopping, household chores) when you thought you would be bleeding?

Never……………………………………………..…………………………………… [ ]

1-3 days……………………………………………………………………………… [ ]

4-8 days……………………………………………...........................………………… [ ]

9-12 days…………………………………………………………………………………... [ ]

13 days or more…………………………….......................………………… [ ]

13. During the past month, when would you carry sanitary products (pads, tampons) with you (in your pocket, in your bag)?

Every day, in case I had any bleeding…………………………………..………………. [ ]

On the days when I had bleeding and on days when I guessed that I might have bleeding [ ]

Only on the days that I had bleeding………………………………………………………. [ ]

14. During the past month, on how many days did you avoid social activities (such as getting together with friends, going shopping for fun, going sight-seeing) when you thought you would be bleeding?

Never……………………………………………..…………………………………… [ ]

1-3 days……………………………………………………………………………… [ ]

4-8 days……………………………………………...........................………………… [ ]

9-12 days…………………………………………………………………………………... [ ]

13 days or more…………………………….......................………………… [ ]

15. During the past month, on how many days did you plan <u>your</u> activities (work, social, or family) based on whether or not there was a bathroom nearby?

Never……………………………………………..…………………………………… [ ]

1-3 days……………………………………………………………………………… [ ]

4-8 days……………………………………………...........................………………… [ ]

9-12 days…………………………………………………………………………………... [ ]

13 days or more…………………………….......................………………… [ ]

© 2025 Rush G et al. *JAMA Network Open*.

16. During the past month, on how many days did you bring extra clothes with you (to work, school, out shopping) in case you had staining from your period?

Never…………………………………………………………………………………………. [ ]

1-3 days………………………………………………………………………………… [ ]

4-6 days………………………………………………………………………………… [ ]

Greater than 6 days……………………………………………………………………... [ ]

17. During the past month, on how many days did you choose what to wear based on whether or not you were bleeding?

Never…………………………………………………………………………………………. [ ]

1-3 days………………………………………………………………………………… [ ]

4-8 days………………………………………………………………………………… [ ]

9-12 days……………………………………………………………………………... [ ]

13 days or more………………………………………………………………………… [ ]

18. On a scale of 0-10, with 0 being no concern at all and 10 being extremely concerned, please rate your overall concern about bleeding staining your clothes.

____

19. During the past month, would you say that your period start date was…

Completely predictable……………………………………………………………... [ ]

Somewhat predictable…………………………………………………………………. [ ]

Not at all predictable……………………………………………………………... [ ]

20. During the past month, would you say that your period end date was…

Completely predictable……………………………………………………………... [ ]

Somewhat predictable…………………………………………………………………. [ ]

Not at all predictable……………………………………………………………... [ ]

© 2025 Rush G et al. *JAMA Network Open*.

eAppendix 2. Adolescent MBQ

Adolescent Menstrual Bleeding Questionnaire
Patient ID # _________
Date of survey _____________(Date/Month/Year)

Instructions:

These questions ask for details about your period. Periods can be different from month to month. Please make sure you read all of the options.

For this questionnaire, period refers to any bleeding that you have from your vagina, even if it is irregular.

Some of the questions may sound similar. Just read through each question carefully and give your best answer.

You may have other medical problems that could affect your answers. Please try to focus on questions and answers ONLY as they relate to your period.

1. What is your current age? _________

2. What grade of school are you currently in? ________

3. What was your age when you got your first period? _____________

4. Average number of days missed from school during the academic year (excluding vacation) due to menstrual bleeding? ______

5. During your most recent period, how would you describe your periods?

a. Very light ______
b. Light _____
c. Moderate _____
d. Heavy ______
e. Very heavy ______

Instructions for questions 6-9:

Tampons or a pad do NOT include “pantyliners”

“Soaked” means completely or almost completely stained and filled with blood.

6. On your heaviest day of bleeding during your most recent period, how many tampons or pads did you soak (either completely or almost completely)?

a. 0 ____
b. 1-4 _____

© 2025 Rush G et al. *JAMA Network Open*.

c. 5-8 _____
d. 9-12 _____
e. 13-16 _____
f. More than 16 _____

7. During your most recent period, how often did you need to wear either an adult diaper or more than one product (either more than one pad, a pad or a tampon, more than one tampon) at a time to contain your bleeding?

a. Never ______
b. 1-3 times ______
c. 4-6 times ______
d. 7-10 times ______
e. 11 times or greater ______

8. During your most recent period, how many times have you had an episode of bleeding that soaked through your "outer" clothes (ie pants/skirt/dress/shorts)?

a. Never ______
b. 1-3 times ______
c. 4-6 times ______
d. Great than 6 times ______

9. During your most recent period, how many times did you need to get out of bed in the middle of the night to change your pad/tampon or adult diaper?

a. Never ______
b. 1-3 times ______
c. 4-6 times ______
d. 7-10 times ______
e. 11 times or greater ______

10. During your most recent period, how many times did you pass blood clots (clumps of blood)?

a. Never ______
b. 1-3 times ______
c. 4-6 times ______
d. Greater than 6 times ______

11. During your most recent period how often did passing blood clots (clumps of blood) stain your clothing?

a. Never ______
b. 1-3 times ______
c. 4-6 times ______
d. Greater than 6 times ______

12. Please fill in the following statement about pain related to your period. During my most recent period, my period was associated with….

a. No pain ______
b. Slight pain ______

© 2025 Rush G et al. *JAMA Network Open*.

c. Moderate pain ______
d. Severe pain ______

13. During your most recent period, how many weeks did your periods last?
a. 1 week or less ______
b. More than one week, less than 2 weeks ______
c. More than 2 weeks, less than 3 weeks ______
d. More than 3 weeks ______

14. During your most recent period, on how many days do you think your school work suffered because you were bleeding?
a. Never, my bleeding does not affect my school work ______
b. 1-3 days ______
c. 4-8 days ______
d. 9-12 days ______
e. 13 days or more ______

15. During your most recent period, how many days of school did you miss because you were bleeding?
a. Never, my bleeding does not affect my school attendance ______
b. Even though I bleed I still go to school ______
c. 1-3 days ______
d. 4-8 days ______
e. 9-12 days ______
f. 13 days or more ______

16. During your most recent period, on how many days did you avoid family activities (chores, shopping) when you thought you were bleeding?
a. Never ______
b. 1-3 days ______
c. 4-8 days ______
d. 9-12 days ______
e. 13 days or more ______

17. During your most recent period, when would you carry sanitary products (pads, tampons) with you (in your pocket, in your bag)
a. Everyday; in case I had any bleeding ______
b. On the days when I had bleeding and on days when I guessed that I might have bleeding ______
c. Only on the days that I had bleeding ______

18. During your most recent period, on how many days did you avoid activities (such as getting together with friends, sleepovers) when you thought you would be bleeding?
a. Never ______
b. 1-3 days ______
c. 4-8 days ______
d. 9-12 days ______

© 2025 Rush G et al. *JAMA Network Open*.

e. 13 days or more ______

19. During your most recent period, on how many days did you plan your activities (school, social or family) based on whether or not there was a bathroom nearby?

a. Never ______
b. 1-3 days ______
c. 4-8 days ______
d. 9-12 days ______
e. 13 days or more ______

20. During your most recent period, on how many days did you bring extra clothes with you (to school, out shopping) in case you had staining from your period?

a. Never ______
b. 1-3 days ______
c. 4-6 days ______
d. Greater than 6 days ______

21. During your most recent period, on how many days did you choose what to wear based on whether or not you were bleeding?

a. Never ______
b. 1-3 days ______
c. 4-8 days ______
d. 9-12 days ______
e. 13 days or more ______

22. On a scale of 0-10, with 0 being no concern at all and 10 being extremely concerned, please rate your overall concern about bleeding staining your clothes __________

23. During your most recent period, would you say that your period start date was

a. Completely predictable ______
b. Somewhat predictable ______
c. Not at all predictable ______

24. During your most recent period would you say that your period end date was

a. Completely predictable ______
b. Somewhat predictable ______
c. Not at all predictable ______

25. Are you bullied or teased at school because of your menstrual period leaking through your outer clothes?

a. Yes ______
b. No ______

© 2025 Rush G et al. *JAMA Network Open*.

eAppendix 3. Short PROMIS, Version 1.2 Physical Health and Short PROMIS, Version 1.2 Mental Health Scales

1. In general, how would you rate your physical health? (check one response)

Excellent ___

Very Good ___

Good___

Fair___

Poor___

2. To what extent are you able to carry out your everyday physical activities such as walking, climbing stairs, carrying groceries, or moving a chair? (check one response)

Completely___

Mostly___

Moderately___

A little___

Not at all___

3. In general, how would you rate your mental health, including your mood and your ability to think? (check one response)

Excellent___

Very Good___

Good___

Fair___

Poor___

4. In general, how would you rate your satisfaction with social activities and relationships? (check one response)

Excellent___

Very Good___

Good___

Fair___

Poor___

© 2025 Rush G et al. *JAMA Network Open*.

eAppendix 4. Patient Survey

The following questions are specifically in relation to sickle cell disease and your menses.

1. Do you experience sickle cell pain around your menstrual cycle menstrual cycle?

   Yes_______No_______

2. **In the past 6 months,** about how many times have you gone to the emergency room (ER) for sickle cell related pain?

   0 times __________________
   1-3 times __________________
   4 or more times______________

3. **In the past 6 months,** about how many times have you been admitted to the hospital for sickle cell related pain?

   0 times __________________
   1-3 times __________________
   4 or more times______________

4. **In the past 2 weeks,** how many days have you taken at least one dose of Ibuprofen (Advil, Motrin, Aleve), Naproxen or Celebrex?

   0 days_____________

   1-4 days ___________

   5 or more days____________

5. **In the past 6 months,** what sickle cell specific medications have you been taking regularly? (Check all that apply)

   i. **Hydroxyurea _____**
   ii. **Crizanlizumab (Adakveo)______**
   iii. **Voxelotor (Oxbryta)_______**
   iv. **L-Glutamine (Endari)_______**
   v. **Chronic blood transfusions________**

6. **Are you on any hormonal therapy also known as Birth Control medication?**

   Yes_______**(skip to question 8)**

© 2025 Rush G et al. *JAMA Network Open*.

No________**(go to question 7)**

7. If you answered NO to question 6, have you heard of hormonal therapy (Birth Control). You do not need to answer question 8.

Yes _________

No__________

8. If you answered YES to Question 6 and you are on hormonal/birth control therapy, please answer the following questions:

a. **In the past 3 months,** what **type** of hormonal/contraceptive therapy are you taking? (check all that apply)
    i. Pill___________
    ii. Injection every 3 months (Depo/shot)________
    iii. Implant under skin (Implanon/Nexplanon)__________
    iv. Intrauterine device (IUD) such as Mirena___, Skyla___, Liletta____, Copper T___ or Paragard___
    v. Barrier (condom)_________

b. **In the past 3 months,** what is the name of your contraceptive medication (if known) ____

c. Why did you choose to start contraceptive therapy?
____
____

d. **For the past 3 months,** what clinic is providing you with prescriptions for this therapy? (check all that apply)
    i. Teen or Adolescent Clinic_____________
    ii. Gynecology clinic______________
    iii. Primary care doctor______________
    iv. Hematologist________________
    v. Other (specify)_________________

© 2025 Rush G et al. *JAMA Network Open*.

eAppendix 5. Provider Survey

Patient ID#:

Date of Provider Survey:

1. **What is the SCD genotype of this patient?**
    a. SS_____
    b. SC______
    c. S beta zero thalassemia______
    d. S beta plus thalassemia________
    e. Other (specify)__________________

2. **Is the patient on Chronic transfusion therapy?**
    Yes (specify below)______No _________
    a. Simple Transfusion ______
    b. Exchange Transfusion _______

3. **How old is this patient?**_____(years)

4. **Current gender**: Male____ Female ______
5. **Gender at birth:** Male_____ Female ______

6. **Patient MBQ Score:** ________

7. **Within the 3 months prior to patient filling out MBQ,** what are the clinic baseline labs for this patient? (Fill only if available in the past 3 months and ensure units are correct)

Test Name	Result	Units
Hemoglobin		g/dL
Mean corpuscular volume (MCV)		fL
Platelet count		TH/mm3
Absolute neutrophil count		TH/mm3
White Blood Cell count		TH/mm3
Reticulocyte percentage		%
Absolute reticulocyte count		MIL/mm3
Total bilirubin		mg/dL
Hemoglobin F		%
Hemoglobin S		%
Hemoglobin A		%
Hemoglobin C (if applicable)		%
Hemoglobin A2 (if available)		%
Hemoglobin other (specify)______		%

© 2025 Rush G et al. *JAMA Network Open*.

8. **Within the 6 months prior to patient filling out MBQ,** has the patient had the following labs? If so, please list results below (ensure units are correct)

Test Name	Result	Units
Ferritin		Ng/mL
Transferrin saturation		%
Total iron binding capacity		Ug/dL
Transferrin level		Mg/dL
Soluble transferrin receptor (sTfR) (if available)		Nmol/L

9. **In the past 6 months**, how many times has this patient been in the ED:

0 times ______

1-3 times ______

4 or more times ____________

10. **In the past 6 months,** how many times has this patient been admitted to the hospital:

0 times ______

1-3 times _______

4 or more times __________

11. **Has the patient had a venous or arterial thrombosis event in the past?**

Yes(answer a,b,c) _____No (skip to next question)_______

a. Venous (vein if available)___________
    i. Central line related? Yes____ No _____
b. Arterial thrombosis including stroke (Location) _____________
c. Unknown (records missing) _____________________

12. **Is the patient currently on any antiplatelet or anticoagulation therapy?**
Yes (specify below) ______No (skip to next question)______

a. Aspirin ______
b. Clopidogrel _________
c. DOAC (specify) ___________
d. Low molecular weight heparin (LMWH) _______________
e. Other (specify) ___________________

13. **In the past 6 months and prior to the MBQ, have you prescribed medications to this patient for heavy menses?**
Yes (specify all that apply below a-d)________

© 2025 Rush G et al. *JAMA Network Open*.

No (skip to next question)______

a. Hormonal therapy (specify) __________
b. Antifibrinolytic therapy (specify) _________
c. NSAIDs (specify) _____________
d. None ________

14. **Based on your assessment of this patient and their MBQ and survey results, what are your next steps? (Check all that apply)**

a. **Referring patient to (apply all that apply):**
   i. Ob/Gyn clinic _____
   ii. Pediatric clinic _______
   iii. Family Medicine clinic _____
   iv. Unknown provider specialty run clinic for women ________

b. **I have the following services available at my institution?**
   i. Ob/Gyn clinic _____
   ii. Pediatric clinic _______
   iii. Family Medicine clinic _____
   iv. Unknown provider specialty clinic for women ____
   v. I do not have any of these clinics available ______

c. **Provided or plan to provide education about contraceptive therapy to patient during current or future clinic visit using (check only if applicable)**
   i. Verbal education _____
   ii. Fliers ______
   iii. Website ______
   iv. Other (specify) _____

d. **Plan to/ Initiated a work up for underlying iron deficiency anemia with ferritin and iron panel**_____

e. **Plan to / Initiated a work up for an underlying bleeding disorder such as factor deficiency, von Willebrand disease, platelet dysfunction**_______

f. **I have no concerns for this patient based on their MBQ score and survey results**
   True / False

g. **Other comments**_____________________

© 2025 Rush G et al. *JAMA Network Open*.

eAppendix 6. Closeout Survey for Participating Sites

1. Does your institution have a combined multidisciplinary hematology (or SCD) and OBGyn clinic?

2. If NO, any plans to start one?

The following are for if YES:

3. Please provide a short description of the clinic (a few words)

4. When was it established?

5. What providers are involved (include social work, psychology, genetic counseling, gynecology, PT, OT, hematologists/SCD specialists, etc. as applicable)

6. How often is this clinic held (weekly, monthly etc.)

7. Is it adult and peds, or just one or the other? (or provide age ranges/groups)

8. How many patients have been seen to date (estimate OK)

9. Most common reason(s) patients are referred? (pregnancy planning, contraception, pain management etc.)

10. Any goals for expansion?

11. Are you collecting patient reports of their main health concerns/feedback about the clinic?

© 2025 Rush G et al. *JAMA Network Open*.

eTable 1. Clinical Laboratory Values Based on Genotype

Genotype	**Overall**	**HbSS**	**HbSβ0**	**HbSC**	**HbSβ+**	**Other**	**P value**
Complete Blood Count (units): * Median							
White blood cell count (Th/mm3): N = 169	9.65	10.15	6.90	10.05	6.80	7.40	<0.01
Hemoglobin (g/dL) N= 173	9.10	8.80	8.80	11.00	10.60	11.60	<0.001
MCV (fL) N= 173	90.00	93.00	94.00	75.50	71.00	82.00	<0.001
Platelet count (Th/mm3): N= 173	369.00	388.00	270.00	302.00	201.00	309.00	<0.05
Absolute neutrophil count (Th/mm3): N = 173	5.04	5.30	2.81	5.12	3.70	3.54	0.107
**Markers of Hemolysis (units): n***							
Total bilirubin (mg/dL) N= 154	1.87	2.20	1.40	1.12	1.30	6.90	<0.001
Reticulocyte count (%) N= 165	7.95	10.20	5.23	4.00	2.29	8.00	<0.001
Absolute reticulocyte (Mil/mm3): N= 126	0.22	0.26	0.14	0.16	0.10	0.04	<0.001
**Hemoglobin Electrophoresis: n***							
Hemoglobin S (%) N= 101	61.90	68.00	64.0	50.45	75.50	NA	<0.05
Hemoglobin A (%) N= 85	13.65	19.60	21.85	0.00	16.50	NA	0.05
Hemoglobin C (%) N= 53	0.00	NA	NA	43.40	NA	NA	<0.001
Hemoglobin A2 (%) N= 68	3.00	2.80	3.40	3.40	5.50	NA	<0.001
Hemoglobin F (%) N = 101	7.60	9.10	26.25	1.70	4.30	NA	<0.001
**Iron Studies (units): n ****							
Ferritin (ng/ml) N= 106	306.00	582.00	564.20	88.50	54.00	6.40	<0.001
Transferrin saturation (%) N= 43	24.00	25.00	39.00	22.50	17.50	NA	0.54
Total iron binding capacity (mcg/dL): N= 45	274.00	254.00	185.00	298.00	254.00	NA	0.14
Transferrin level (mg/dL) N= 16	169.00	169.00	0.00	270.00	0.00	NA	0.07

*Included only if collected within 3 months prior to enrollment

**Included only if collected within 6 months prior to enrollment

© 2025 Rush G et al. *JAMA Network Open*.

eTable 2. Site-Specific Multidisciplinary Clinic Services

Site	Date established	Services in clinic	Frequency	Notes
1	2013	• Hematology<br>• Gynecology	Twice monthly	Pediatric and young adult
2	2020	• Hematology<br>• Adolescent medicine<br>• Gynecology<br>• Nurse educator<br>• Psychology<br>• Genetic counselor<br>• Physical therapy<br>• Social work	Monthly	Pediatric<br><br>Plan to improve transition to adult care
3	2022	• Hematology<br>• Adolescent medicine<br>• Gynecology<br>• Nurse coordinator<br>• Social work	Monthly	Pediatric and young adult<br><br>Plan to add adult multidisciplinary clinic
4	2023*	• Hematology<br>• Gynecology<br>• Maternal-fetal medicine<br>• Nurse coordinator<br>• Psychology<br>• Genetic counseling<br>• Social work	Quarterly	Pediatric, young adult, adult
5	2023*	• Hematology (pediatric)<br>• Adolescent medicine<br>• Social work<br>• Psychology<br>• Genetic counseling	Monthly	Pediatric
6	2014; 2024*	• Hematology (pediatric and young adult)<br>• Gynecology<br>• Adolescent medicine<br>• Nurse	Monthly	Pediatric and young adult<br><br>A combined hematology-gynecology clinic was opened in 2014 for all bleeding disorders; in 2024 a SCD-specific clinic was started.

*Established during study enrollment period

© 2025 Rush G et al. *JAMA Network Open*.
