# Supplementary material for: Role of Menstrual Bleeding Assessments in Sickle Cell Clinics
Source: JAMA Netw Open. 2025 Dec 9;8(12):e2546345. doi: 10.1001/jamanetworkopen.2025.46345 (PMC12690426; doi:10.1001/jamanetworkopen.2025.46345)
Supplement: Supplement 2. — Data Sharing Statement [file jamanetwopen-e2546345-s002.pdf]

Data Sharing Statement

Data

Data available: Yes
Data types: Deidentified participant data, Data dictionary
How to access data: neha.bhasin@ucsf.edu
When available: With publication

Supporting Documents

Document types: None

Additional Information

Who can access the data: Anyone requesting the data
Types of analyses: For any purpose
Mechanisms of data availability: With investigator support
Any additional restrictions: Please contact neha.bhasin@ucsf.edu to request study data.
